# Lightweight-Convolutional Neural Network for Apple Leaf Disease Identification

**DOI:** 10.3389/fpls.2022.831219

**Published:** 2022-05-24

**Authors:** Lili Fu, Shijun Li, Yu Sun, Ye Mu, Tianli Hu, He Gong

**Affiliations:** ^1^College of Information Technology, Jilin Agricultural University, Changchun, China; ^2^College of Electronic and Information Engineering, Wuzhou University, Wuzhou, China; ^3^Jilin Province Agricultural Internet of Things Technology Collaborative Innovation Center, Changchun, China; ^4^Jilin Province Intelligent Environmental Engineering Research Center, Changchun, China; ^5^Jilin Province Information Technology and Intelligent Agricultural Engineering Research Center, Changchun, China

**Keywords:** lightweight, multi-scale, attention, cavity convolution, apple leaf disease

## Abstract

As a widely consumed fruit worldwide, it is extremely important to prevent and control disease in apple trees. In this research, we designed convolutional neural networks (CNNs) for five diseases that affect apple tree leaves based on the AlexNet model. First, the coarse-grained features of the disease are extracted in the model using dilated convolution, which helps to maintain a large receptive field while reducing the number of parameters. The parallel convolution module is added to extract leaf disease features at multiple scales. Subsequently, the series 3 × 3 convolutions shortcut connection allows the model to deal with additional nonlinearities. Further, the attention mechanism is added to all aggregated output modules to better fit channel features and reduce the impact of a complex background on the model performance. Finally, the two fully connected layers are replaced by global pooling to reduce the number of model parameters, to ensure that the features are not lost. The final recognition accuracy of the model is 97.36%, and the size of the model is 5.87 MB. In comparison with five other models, our model design is reasonable and has good robustness; further, the results show that the proposed model is lightweight and can identify apple leaf diseases with high accuracy.

## Introduction

As one of the most widely consumed fruits, apple plays a vital role in economic development and meeting people’s daily needs. However, faced with a complex and ever-changing natural environment, the growth process of apples is affected by a variety of diseases, which will have a large impact on their yield and quality (Sethy et al., 2020). Therefore, strengthening our ability to control apple leaf diseases is imperative to ensure apple yield and quality. Quickly and accurately detecting such diseases, evaluating them, and then effectively controlling them can help to inform the rational use of agricultural resources such as pesticides, fertilizers, and water for apple growth. At present, the traditional method for the discrimination of plant diseases mainly relies on recognition by vision, which requires the discriminator to have sufficient experience in the field. Further, subjective assumptions also affect the accuracy of discrimination, and this method cannot be used to quantify disease identification. Owing to the complex symptoms of apple leaf diseases, incorrect evaluations can lead to an overuse of pesticides by fruit farmers, which not only fails to prevent and control the disease but also leads to environmental issues (Wu, 2015). With the rapid development of artificial intelligence, CNNs are widely used to solve issues regarding plant diseases, and scholars conducted significant research on this topic.

Radha (2017) preprocessed images through image loading, contrast enhancement, and RGB-HSI conversion, using support vector machine (SVM) as a classifier to identify a variety of plant diseases. Lin et al. (2019) constructed a semantic segmentation model to segment the disease spots of cucumber powdery mildew, and the final accuracy reached 82.19%. Sharma et al. (2020) trained an S-CNN model that was able to accurately identify nine tomato leaf diseases after segmentation, with an accuracy of 98.6%. Xie et al. (2019) used a morphological expansion algorithm to remove noise from images and classify features using SVMs to extract color and texture feature information from tobacco images more efficiently. Pandiyan et al. (2020) constructed a platform for plant leaf disease identification and prediction analysis, which used an advanced segmented dimensional approach to extract plant leaf disease features and achieved real-time identification and analysis capabilities. Zhang et al. (2020) constructed a two-level classification model for similar diseases in cucumbers using an efficient network—B4—and a state-of-the-art optimizer—Ranger—to identify and classify two diseases on greenhouse cucumber leaves, obtaining an accuracy of 96%. Jiang et al. (2020) combined the deep learning and SVM techniques to identify and classify rice diseases, and the average correct recognition rate of the model was 96.8%. Bhagwat and Dandawate (2021) proposed a multi-scale CNN for plant disease detection and conducted comparative experiments with the currently popular CNNs, exhibiting the better robustness of their method in processing various types of leaf images. Huang et al. (2020) constructed a segmentation model based on the DeepLabV3 model, with a deep separable residual network (ResNet) as the backbone network, naming it “Apple Net.” Their model could effectively segment apples and apple leaves. Sardogan et al. (2020) constructed an Incept-Faster-RCNN model, which introduced the Inception v2 structure to the Faster-RCNN model for the detection of two apple leaf diseases; its detection accuracy was 84.50%. Wang et al. (2021) proposed a deep learning model (CA-ENet) for identifying apple diseases, which applied deep separable convolution to reduce the number of parameters and obtained good recognition results.

However, some researchers have used too many parameters for their model to be practically applied (Boulent et al., 2019). In this regard, some scholars have proposed lightweight CNN models for plant disease identification. Rahman et al. (2020) proposed a two-level small CNN architecture for identifying rice pests and diseases, to address the issue whereby large-scale architectures are not suitable for mobile devices, and experimental results showed that the proposed architecture could achieve 93.3% of the expected accuracy with a significantly reduced model size. Gonzalez-Huitron et al. (2021) introduced deep separable convolution in a convolutional model to make the model more lightweight, then trained the model with publicly available datasets, and finally successfully implemented the lightweight architecture model on a Raspberry Pi 4 microcomputer. Bao et al. (2021) designed a Simple Net CNN model to identify barley spike disease, using which the spatial attention module extracts the important features and reduces the influence of complex backgrounds on the recognition effect; the final model had 2.13 million parameters, with a 94.1% recognition accuracy.

In previous research by scholars, lightweight characteristics and a high recognition accuracy of CNN models most often could not be achieved simultaneously. To mitigate this issue, inspired by previous studies, we propose a new lightweight CNN model with AlexNet as the base network and apple leaf disease as the recognition object. The main contributions of this study are summarized as follows:

1. Parallel convolution was used to extract disease features from leaves with complex backgrounds to improve the model recognition accuracy; dilated convolution is introduced into the model to reduce the number of parameters while maintaining the sensory field.
2. Channel attention was embedded in the aggregation module of the model to extract more important disease features and reduce the impact of complex backgrounds on model performance; the final model is lightweight and achieves a high recognition accuracy.

The remainder of the study is organized as follows: section “Materials and Methods” provides an overview of the sources and processing of the dataset, describing the principles, as well as the advantages and disadvantages, of the classical network AlexNet. Section “Apple Leaf Disease Identification Modeling” describes, in detail, the formulation and improvements in the model before evaluating the model performance in section “Results and Analysis.” Section “Conclusion” summarizes the findings of this study and provides future perspectives.

## Materials and Methods

### Image Data and Processing

**FIGURE 1:**
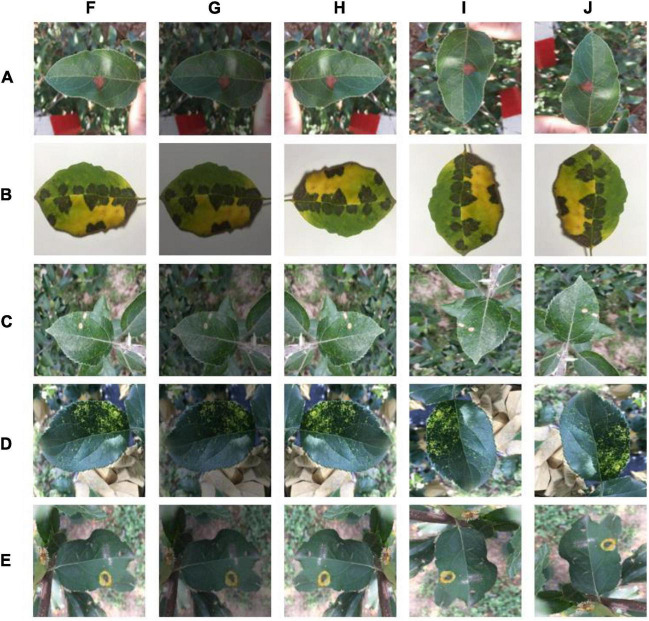
Disease types and selected image enhancement methods: **(A)** Alternaria blotch, **(B)** brown spot, **(C)** gray spot, **(D)** mosaic, **(E)** rust, **(F)** original, **(G)** color dithering, **(H)** mirror flip, **(I)** 270° rotation, and **(J)** 90° rotation.

In the field of plant disease research, many scholars use the publicly available PlantVillage dataset to train models (Mohanty et al., 2016; Ferentinos, 2018; Chohan et al., 2020; Atila et al., 2021). This dataset contains simple background images (Hughes and Salathé, 2015). The experimental data used in this experiment are from the publicly available AI Studio dataset. This dataset includes not only images with simple backgrounds, but also images with complex backgrounds. The types of apple leaf diseases and their numbers are shown in Table 1, including mosaic, brown spot, gray spot, spotted leaf drop, and rust. The dataset is complex and diverse, with all four diseases containing complex background images, except for brown spots, which have simple background data. The five disease images and some of the image enhancement methods used are shown in Figure 1. A total of 26,377 sample images were collated from the dataset, and the dataset images were compressed to a uniform size of 224 × 224 using an interpolation algorithm. The sample data were randomly divided into a training set, validation set, and test set, at a ratio of 7:2:1 using Python, with 18,466 images in the training set, 5,274 images in the validation set, and 2,637 images in the test set.

**TABLE 1:** Apple leaf disease classes and the amount of data in different contexts.

Class	Image background	Number of images	Total
Alternaria blotch	Simple	1,755	5,343
	Complex	3,588	
Brown spot	Simple	5,655	5,655
	Complex	0	
Gray spot	Simple	2,288	4,810
	Complex	2,522	
Mosaic	Simple	2,795	4,875
	Complex	2,080	
Rust	Simple	5,473	5,681
	Complex	208	

### Strengths and Weaknesses of the Original AlexNet

**FIGURE 2:**
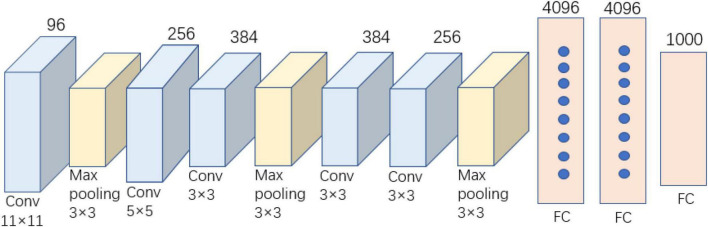
Sketch of the AlexNet model structure.

AlexNet was published in 2012 and won the annual ILSVRC competition owing to its significant advantages (Krizhevsky et al., 2012). The AlexNet network requires an input image size of 224 × 224 × 3, with a total of eight layers. The structure of our simplified AlexNet model is shown in Figure 2, including five convolutional layers, three pooling layers, and three fully connected layers. AlexNet, the first CNN model used for image recognition and classification, has the characteristics of local connection, weight sharing, and pooling. Its structure is relatively simple, and it does not require complex hardware equipment for the model architecture. Many of the techniques used in AlexNet provide the basis for the industrial application of CNNs. However, AlexNet’s entire network model contains 630 million connections, 60 million parameters, and nearly 650,000 neurons (Guo et al., 2019). The final model size was approximately 222.4 MB, which severely limited the training time and speed of the model. Further, the use of several parameters makes the model highly susceptible to overfitting during training.

## Apple Leaf Disease Identification Modeling

This research model improves upon the original AlexNet model in terms of its shortcomings: a large number of parameters and a low recognition accuracy. The improved model structure is relatively simpler than that of other CNN models, and its performance can also reach the desired level. The structure of the apple leaf disease identification model built in this study is as follows.

### Increasing the Model Receptive Field

**FIGURE 3:**
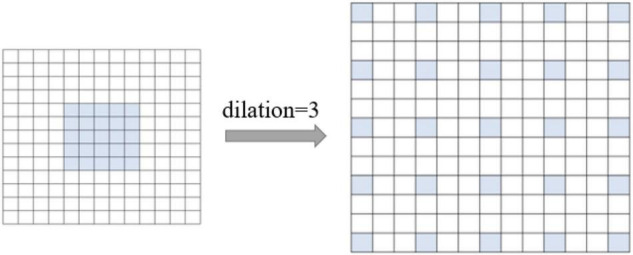
Dilated convolution.

The first layer of AlexNet is a large 11 × 11 convolutional kernel, which has a large range of receptive fields but increases the number of model parameters—to a certain extent—and limits the speed of the model. Therefore, our model uses a small 5 × 5 convolution instead of a large 11 × 11 convolution kernel, converting the small 5 × 5 convolution into a 13 × 13 dilated convolution by setting its dilation to 3. As shown in Figure 3, the convolution has a large receptive field of 13 × 13, while requiring only the number of parameters of a 5 × 5 convolution, increasing the computation speed.

### Multi-Scale Extraction of Disease Features

We added a multi-scale convolution module to the model because of the large number of apple leaf disease features and their widespread distribution locations on the leaves. A number of four convolutions—1 × 1, 3 × 3, 5 × 5, and 7 × 7—are connected in parallel, and the output feature maps are stitched together using the “depthconcat” operation to fuse them into a disease feature map with more features. The sequence and excitation network (SE) module is used to fit the value of the channel of the characteristic diagram; the calculation process of SE is shown in Equations 1, 2. The feature map is first compressed into a 1 × 1 × C size feature map by global average pooling (F_*i*_) with Squeeze. Excitation is then performed: a nonlinear transformation of the result after squeezing is first performed using a fully connected neural network (σ), after which Elu, fully connected (σ), and sigmoid operations are performed sequentially to obtain CW_*i*_. This is finally fused with the original feature (F_*i*_) to obtain the feature map (CF_*i*_). This operation can extract the more important features, while using multi-scale convolution to extract features can maximize the retention of feature points and improve the recognition accuracy of the model.


(1) $$\text{C}\,W_{i}=\text{s}\,i\,g\,m\,o\,i\,d\,\left(\sigma \,\left(\text{E}\,l\,u\,\left(\sigma \,\left(\varphi \,\left({\text{F}}_{i}\right)\right)\right)\right)\right)$$

(2) $$\text{C}\,F_{i}=\text{C}\,W_{i}\cdot {\text{F}}_{i}$$

### Shortcut Connection Enhances Model Performance

**FIGURE 4:**
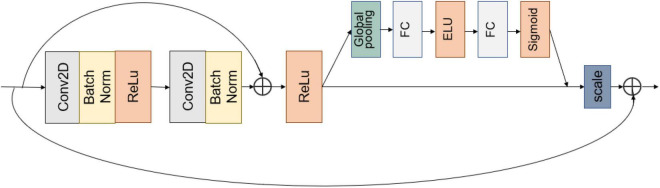
BasicBlock + SE module.

Convolutional blocks in series increase the depth of the model. However, blindly increasing the depth of the model without considering other factors lowers the model performance (Tan and Le, 2019). Network accuracy can become saturated and even degrade when the network depth increases (He et al., 2016). This model takes two 3 × 3 convolutions and forms them into a BasicBlock module using a shortcut connection, as shown in Figure 4. By aggregating features, the model learns disease features more easily and directly, effectively mitigating the disappearance of model gradients.

**FIGURE 5:**
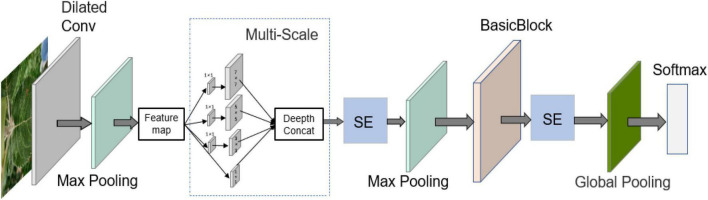
Apple leaf disease identification model.

The structure of the model constructed for this study is shown in Figure 5, and the specific parameters of each module in the structure are shown in Table 2. When building the network, the ReLU activation function is added after all convolutional layers to increase the model nonlinearity and ensure that the model gradient decreases. In addition, the local response normalization layer (LRN) is removed entirely and replaced with a batch normalization layer (BN) to speed up the forward and backward propagation rates of the model. Research, in the recent years, has shown that the efficient design of the network structure can reduce the number of parameters in the model by simplifying the fully connected layers (Ioffe and Szegedy, 2015; Szegedy et al., 2015, 2016, 2017). The two fully connected layers of AlexNet are replaced by a global pooling structure, which guarantees the accuracy of the model while significantly reducing the number of parameters.

**TABLE 2:** Table of module parameters in our model.

Layer	Size		Output
Input	224 × 224		
Dilated convolution	13 × 13		96 × 109 × 109
Max pooling	3 × 3		96 × 28 × 28
Multi-scale	1 × 1		64 × 28 × 28
	1 × 1	3 × 3	128 × 28 × 28
	1 × 1	5 × 5	32 × 28 × 28
	1 × 1	7 × 7	32 × 28 × 28
SE module	(256, 16)		256 × 28 × 28
Max pooling	3 × 3		256 × 14 × 14
BasicBlock	3 × 3		256 × 14 × 14
	3 × 3		256 × 14 × 14
SE module	(256, 16)		256 × 14 × 14
Global pooling			
Softmax			

## Results and Analysis

### Experimental Environment and Parameter Settings

The deep learning framework used in this experiment was Pytorch 1.8.0. The version of Torchvision was 2.2.4 and the computer configuration was an Intel Core i7-8700 CPU running at 3.20 GHz. It was equipped with an Nvidia GeForce GTX 1080 graphics card. The adapter was an Intel UHD Graphics 630. The software was CUDA API version 0.9.10, based on the Python 3.8.3 programming language and integrated with the PyCharm2020 development environment.

To better assess the difference between the true and predicted values, the hyperparameters were set as shown in Table 3. In this study, the training and validation process is divided into multiple batches using the batch-training method, and the loss function is adopted as cross-entropy loss. In addition, the weight initialization method is set to Xavier and the initialization bias is set to 0. Finally, the softmax classifier is used, and all models are trained from scratch.

**TABLE 3:** Hyperparameter settings.

Hyperparameters	Values
Classes	5
Batch size	32
Epochs	40
Optimizer	Adam
Learning rate	0.001
Momentum	0.9

### Experimental Results and Analysis

**FIGURE 6:**
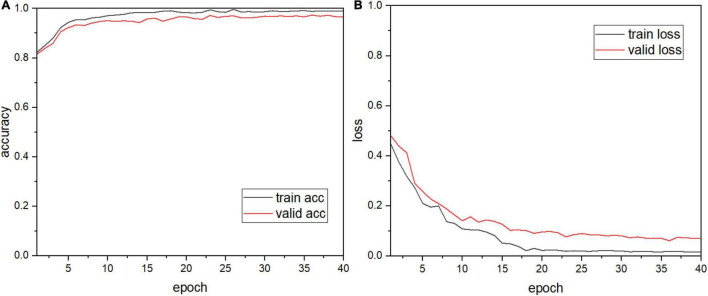
Our model graph: **(A)** accuracy and **(B)** loss.

The apple leaf disease recognition model was trained according to the algorithm and recognition process proposed above. First, the process of building the model and the modules were explored, the results of which are shown in Table 4. Since the number of parameters of the fully connected layers is the largest in the entire model, global pooling was first used to replace the fully connected layers [model (1)]. It can be seen that the number of parameters of the model is greatly reduced and the size of the model is reduced from 222.4M to 14.54M. Based on this, injection dilated convolution is added to obtain model (2), which effectively reduces the number of model parameters. Model (3) adds a multi-scale convolution module to model (2) and makes full use of its parallel convolution function to reduce the size of the model again. As shown in the table, model (4) implements a shortcut connection to model (3). After convoluting the last three layers of the original AlexNet model and discarding one layer, the remaining two layers are connected through shortcut connection. It can be seen that there are obvious changes in the number of model parameters and model size. Finally, the SE module is introduced behind the aggregate output module of model (4) to complete the construction of the model. The table shows that the parameter size of our model is 5.86M, and the number of parameters is only 1/38 that of AlexNet, which proves that the model built in this study is accurate and feasible. The plot of the model recognition rate and the loss rate is shown in Figure 6. As can be seen from the figure, our model converges quickly and the model accuracy improves dramatically, exceeding 90% at the fourth epoch. The introduction of the SE module adds more nonlinearity to the model, making it more stable, and it plateaus at 15 epochs. The recognition accuracy of the final verification of the model in this study is 97.36%, and the average loss rate of the verification is 0.1396.

**TABLE 4:** Comparison of the parameters of each module: (1) add global pooling, (2) add dilated convolution, (3) add multi-scale convolution, and (4) use shortcut connection.

Model	Global pooling	Dilated convolution	Multi-scale	Shortcut connection	SE	Total parameters	Parameter size
AlexNet						58,301,829	222.4
(1)	√					3,812,677	14.54
(2)	√	√				3,784,933	14.44
(3)	√	√	√			3,337,829	12.37
(4)	√	√	√	√		2,336,101	8.91
Ours	√	√	√	√	√	1,535,579	5.86

### Model Performance Validation

**FIGURE 7:**
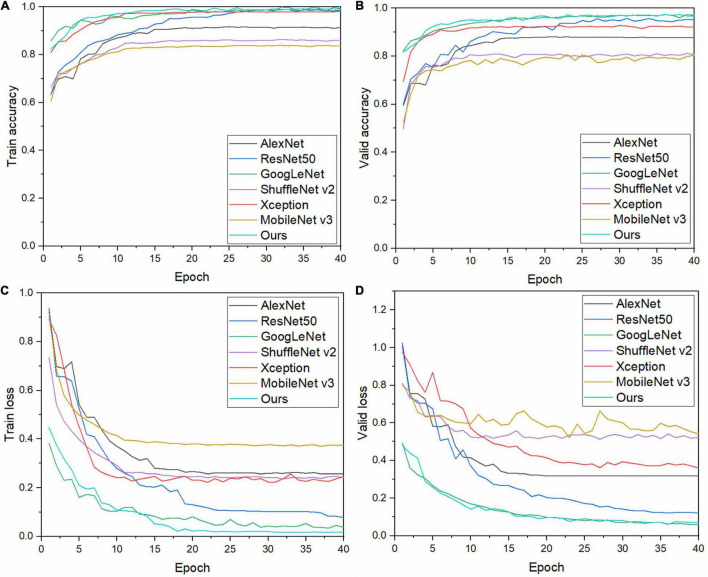
Training and validation results of each model: **(A)** training accuracy, **(B)** validation accuracy, **(C)** training loss, and **(D)** validation loss.

To verify the performance of the model, its performance is compared with those of six other CNN models. Keeping the abovementioned experimental method and experimental environment unchanged, each model is trained, the results of which are shown in Figure 7. Our model has a high initial recognition accuracy, low loss rate, and fast convergence. Figures 7A,B show that the curves of our model are relatively smooth and have fewer fluctuations than those of other models during both training and validation, which indicates that our model exhibits good stability.

Keeping the training method and training parameters unchanged, the results of each model listed in Table 5 are obtained. Among them, the recognition accuracy of AlexNet, ResNet50, GoogleNet, and Xception for apple leaf diseases is low, whereas the parameter sizes of the models are large, and the performances are lower than that of our model. The parameter size of MobileNet V3 is the smallest. This is because MobileNet V3 uses NAS technology to automatically optimize the model with ImageNet as the dataset (Howard et al., 2019), which greatly reduces the number of parameters of the model; however, the universality of the model is not high. In terms of recognition accuracy, our model recognition accuracy is 97.36%, 16% points higher than that of MobileNet V3. The floating point of operations (flops) of our model is 2.553G, which is larger than those of ShuffleNet V2 and MobileNet V3. This is because we add a multi-scale convolution module to our model, which improves the accuracy of model recognition and reduces the number of parameters required; however, this leads to cross-calculation between the parameters of the model, resulting in a high calculation time. Similar to Xception, which is a multi-scale structure, the model itself is lightweight, but the number of flops is 4.58 g. In addition, using the size of the flops alone cannot objectively measure whether the model is lightweight (Ma et al., 2018); this metric is ascertained in combination with the recognition time. As can be seen from the table, the recognition time of our model is 0.2229 (s), which is less than that of ShuffleNet V2 and MobileNet V3. In conclusion, it can be seen that the lightweight CNN model constructed in this study is reasonable, with a high recognition accuracy and a lower number of model parameters. This will make the identification of apple leaf diseases in an actual agricultural environment faster, reducing identification time and control costs, and better meeting the application needs in actual complex agricultural environments.

**TABLE 5:** Performance comparison of each model.

Architecture	Validation accuracy (%)	Parameter size (MB)	FLOPs	Time
AlexNet	88.05	222.4	711.5M	0.2560
ResNet50	95.66	89.72	4.12G	0.2310
GoogLeNet	97.25	22.81	2G	0.2088
ShuffleNet V2	81.13	5.26	591.8M	0.3068
Xception	92.75	20.82	4.58G	0.3485
MobileNet V3	80.64	2.68	262.12M	0.2270
Ours	97.36	5.87	2.553G	0.2229

### Model Evaluation

**FIGURE 8:**
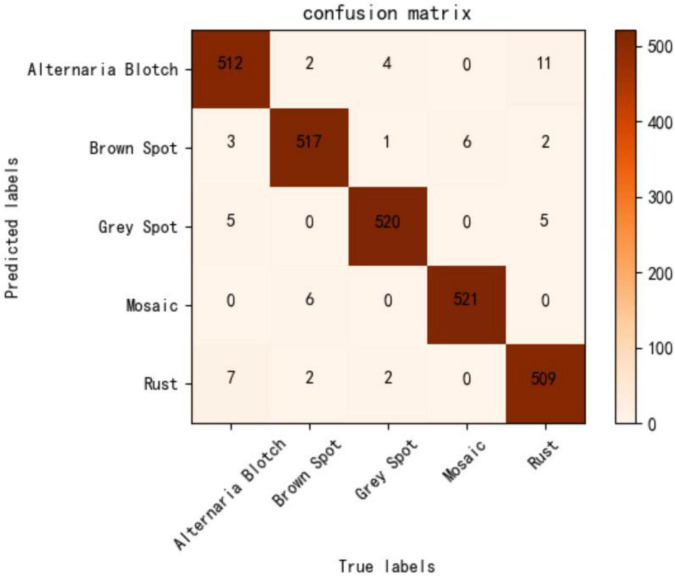
Confusion matrix of our model.

To evaluate the performance of the proposed method objectively and comprehensively, the test set is used to test the performance of the model, and the results are displayed intuitively using a confusion matrix. Figure 8 visually shows the classification performance of the lightweight model constructed in this study. Misclassification mainly occurred between Alternaria blotch, rust, and gray spots, all of which appear as small patches on the leaves, with only slight differences in their color and shape. Second, both brown spots and mosaic spots are densely distributed on the leaves over a large area, leading to the misclassification of disease categories. It can be seen that the performance of our model in mitigating the issue of similarity between disease spots must be improved. However, the number of false positives in the model is still within acceptable limits.

**FIGURE 9:**
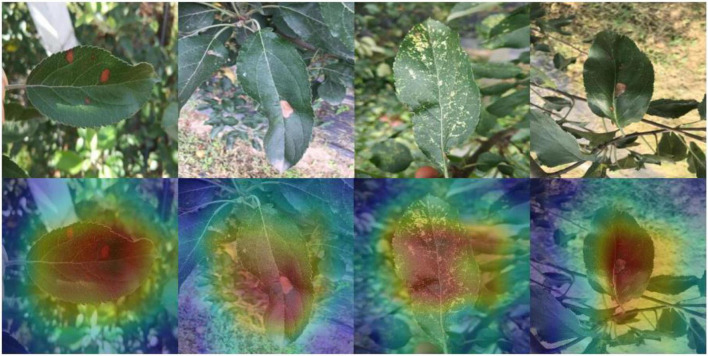
Heat map of our model: the disease area of apple leaves is shown with a certain brightness.

Because images with complex backgrounds are present in the dataset used for this study, to avoid classification bias, the model identification process is visualized. The important information in each channel is multiplied by the convolution activation value, and a thermal map is generated according to the product value, which is superimposed on the original map to obtain the thermal map of apple leaf disease. As shown in Figure 9, the dark colors are mostly focused on the disease rather than the background, demonstrating the effectiveness of our model in identifying data in complex scenarios.

## Conclusion

To further improve the control of apple diseases and explore new methods for apple leaf disease identification, a novel method is proposed herein. This method has stronger practical application capabilities to promote the development of intelligent management methods. In this study, the model was used to extract disease spot features to identify apple leaf diseases by utilizing deep learning to build an apple leaf disease identification model based on the large network, AlexNet. In our model, coarse-grained features are extracted using dilated convolution to reduce the number of model parameters. Based on this, a multi-scale extraction module is added to improve the recognition accuracy of the model. Furthermore, a shortcut connection is constructed to make the model more nonlinear. Finally, the LRN is removed, the BN layer is added, and the fully connected layer is replaced by global pooling, which not only increases the accuracy of model recognition, but also greatly reduces the number of model parameters. The recognition accuracy of the lightweight model constructed in this study is 97.36%, and the parameter size of the model is only 5.87 MB. Comparing the results of five other network models shows that the performance of our model can reach an optimum value. The model was evaluated with a test set; the final confusion matrix and heat map demonstrated the effectiveness of our model for apple leaf disease identification. This method provides a reference for further research on lightweight apple disease identification methods and offers more possibilities for the practical application of apple leaf disease identification in complex backgrounds in real environments.

## Data Availability Statement

The original contributions presented in the study are included in the article/supplementary material, further inquiries can be directed to the corresponding author.

## Author Contributions

LF designed and performed the experiment, selected the algorithm, analyzed the data, trained the algorithms, and wrote the manuscript. YM and TH conceived the study. SL, HG, and YS gave guidance to this research. All authors contributed to the article and approved the submitted version.

## Conflict of Interest

The authors declare that the research was conducted in the absence of any commercial or financial relationships that could be construed as a potential conflict of interest.

## Publisher’s Note

All claims expressed in this article are solely those of the authors and do not necessarily represent those of their affiliated organizations, or those of the publisher, the editors and the reviewers. Any product that may be evaluated in this article, or claim that may be made by its manufacturer, is not guaranteed or endorsed by the publisher.
